# A qualitative analysis exploring preferred methods of peer support to encourage adherence to a Mediterranean diet in a Northern European population at high risk of cardiovascular disease

**DOI:** 10.1186/s12889-018-5078-5

**Published:** 2018-02-05

**Authors:** Christina M. Erwin, Claire T. McEvoy, Sarah E. Moore, Lindsay Prior, Julia Lawton, Frank Kee, Margaret E. Cupples, Ian S. Young, Katherine Appleton, Michelle C. McKinley, Jayne V. Woodside

**Affiliations:** 1 Centre for Public Health, Queen’s University Belfast, Grosvenor Road, Belfast, BT12 6BJ UK; 2 UK Clinical Research Collaboration Centre of Excellence for Public Health, Queen’s University Belfast, Grosvenor Road, Belfast, BT12 6BJ UK; 3 Centre for Population Health Sciences, University of Edinburgh Medical School, Teviot Place, Edinburgh, EH8 9AG UK; 4 Department of Psychology, Bournemouth University, Fern Barrow, Talbot Campus, Poole, Dorset, Bournemouth, BH12 5BB UK

**Keywords:** Peer support, Behaviour change, Mediterranean diet, Cardiovascular disease

## Abstract

**Background:**

Epidemiological and randomised controlled trial evidence demonstrates that adherence to a Mediterranean diet (MD) can reduce cardiovascular disease (CVD) risk. However, methods used to support dietary change have been intensive and expensive. Peer support has been suggested as a possible cost-effective method to encourage adherence to a MD in at risk populations, although development of such a programme has not been explored. The purpose of this study was to use mixed-methods to determine the preferred peer support approach to encourage adherence to a MD.

**Methods:**

Qualitative (focus groups) and quantitative methods (questionnaire and preference scoring sheet) were used to determine preferred methods of peer support. Sixty-seven high CVD risk participants took part in 12 focus groups (60% female, mean age 64 years) and completed a questionnaire and preference scoring sheet. Focus group data were transcribed and thematically analysed.

**Results:**

The mean preference score (1 being most preferred and 5 being least preferred) for group support was 1.5, compared to 3.4 for peer mentorship, 4.0 for telephone peer support and 4.0 for internet peer support.

Three key themes were identified from the transcripts:

1. Components of an effective peer support group: discussions around group peer support were predominantly positive. It was suggested that an effective group develops from people who consider themselves similar to each other meeting face-to-face, leading to the development of a group identity that embraces trust and honesty.
2. Catalysing Motivation: participants discussed that a group peer support model could facilitate interpersonal motivations including encouragement, competitiveness and accountability.
3. Stepping Stones of Change: participants conceptualised change as a process, and discussed that, throughout the process, different models of peer support might be more or less useful.

**Conclusion:**

A group-based approach was the preferred method of peer support to encourage a population at high risk of CVD to adhere to a MD. This finding should be recognised in the development of interventions to encourage adoption of a MD in a Northern European population.

## Background

Cardiovascular disease (CVD) is currently a leading cause of morbidity and mortality worldwide and its incidence is growing rapidly in low and middle income countries [1]. Epidemiological studies demonstrate that adoption and adherence to a Mediterranean diet (MD) can help to prevent CVD. A meta-analysis of cohort studies found that a 2-point increase in adherence to a MD was significantly associated with an 8% reduction in all-cause mortality, a 10% decrease in incidence of cardiovascular and cerebrovascular diseases, a 6% reduction in incidence of neoplastic diseases and a 13% decrease in incidence of neurodegenerative diseases [2]. The PREDIMED randomised control trial (RCT) found that an intensive intervention to encourage adherence to a MD resulted in a significant 30% reduction in CVD risk over 5 years [3]. The intensive methods employed in the PREDIMED study allowed demonstration of efficacy in relation to CVD prevention but the cost of this approach makes it prohibitive to scale up to a population level.

Epidemiological evidence also suggests that adherence to a MD is associated with a reduced risk of developing type 2 diabetes [4–7]. The MD has been shown, in RCTs, to improve glycaemic control in diabetic populations [7–14], and to reduce the need for anti-hyperglycaemic drug therapy in overweight patients with newly diagnosed type 2 diabetes [15].

The MD places emphasis upon a high intake of fruits, vegetables, wholegrain cereals, beans, nuts and seeds. It includes olive oil as a major fat source and dairy products, fish and poultry are consumed in low to moderate amounts, eggs are consumed zero to four times weekly and little red meat is consumed [16]. It has been proposed to be an alternative, palatable, beneficial lifestyle change [17].

Peer support offers a means of delivering social support, knowledge, skills and resources to people in order to improve self-efficacy and help encourage positive lifestyle changes that can reduce risk of CVD. Furthermore, some studies suggest that *providing* support to others may provide comparable, or possibly greater, health and other benefits than receiving support [18, 19].

Peer support has been defined as “the provision of emotional, appraisal, and informational assistance by a created social network member who possesses experiential knowledge of a specific behaviour or stressor and similar characteristics as the target population, to address a health-related issue of a potentially or actually stressed focal person” [19].

A peer support intervention presents the prospect of a mutually-beneficial, less resource intensive method of encouraging lifestyle changes that can contribute to the prevention of chronic disease. There are many different peer support approaches available, including face-to-face in a group or one-to-one format, telephone-based peer support, and web and e-mail-based peer support. Gibson [20] recommends that studies engage with the public to identify the most acceptable approach to peer support for the population and the intended outcome. It has been suggested that for peer support programmes to be effective they should be culturally sensitive and tailored to the target population [21, 22]. Furthermore, there are specific aspects of peer support interventions that could contribute to the effectiveness of such programmes, including the identification of characteristics of ideal peer supporters, that have not been explored in detail [22].

The research presented here examines preferred methods for peer support amongst people at high risk of CVD to encourage them to adopt and maintain a MD. This work will inform the design of an intervention to support the adoption of a MD, with the aim of testing its effectiveness in a randomised controlled trial in line with the MRC framework [23, 24].

## Methods

Focus groups were chosen to investigate the acceptability of different peer support interventions and to explore the skills and characteristics necessary in peer supporters. Focus groups were preferred to other methods of data collection, such as interviews, to allow access to group norms rather than personal views.

The study was carried out in Northern Ireland and was approved by the Office for Research Ethics Committees Northern Ireland (ORECNI) (Registration 12/NI/0043). Focus groups were conducted by two researchers (CMcE and SM) at community centres, general practices and at the Centre for Public Health, Queen’s University Belfast. The transcripts were analysed by a third researcher (CE), who was not involved in the data collection.

### Recruitment and eligibility

Participants were recruited from general practices via posters, through contact with community groups and networks, and identified from hospital outpatient clinic lists, which were scanned for suitable participants, who were contacted by letter to ask them whether they would like to opt in to study participation. Former participants in dietary intervention studies at the Centre for Public Health, Queen’s University Belfast, who had consented to future contact, were also invited to participate via telephone. Participants were eligible if they were aged over 50 and had two or more specified risk factors for CVD (overweight or obese, smoking, hypertension and hypercholesterolemia). Participants were excluded if they had existing CVD or type 2 diabetes.

Twelve focus groups were held between December 2012 and July 2013 with between 2 and 11 participants in each. This number of groups facilitated the recruitment of a diverse study population allowing us to access a diverse range of perspectives and achieve data saturation. Each focus group was similar in socioeconomic status and homogenous in gender, this was considered important to facilitate uninhibited discussion.

Focus group sampling was purposive, to ensure that people of different ages, genders, and from different socio-economic, domestic and geographical settings took part.

### Quantitative data collection

Prior to each focus group, demographic and dietary information (8-item MD food frequency questionnaire) was collected from all participants in a brief questionnaire. The MD questionnaire was developed based on a validated 14-item MDS questionnaire [25] and current guidance for MD consumption [26]. Points were allocated for high consumption of olive oil, fruit, vegetables, oily fish, wholegrains or nuts, moderate consumption of wine or low intake of red meat. Two forms were provided at the conclusion of each focus group, one for participants to indicate their personal preferred method of peer support and the other to indicate their personal preferences regarding peer supporter characteristics.

### Quantitative data analysis

Information from the questionnaire was entered into IBM SPSS Statistics (v 21) and was analysed using descriptive statistics (Table 1). Preference for peer support approach was examined for all participants, and by gender (Table 2). Preference for peer supporter characteristics was collated (Table 3).

**Table 1:** Participant Characteristics summarised for each focus group

Focus Group	Number attending	Gender	Geographical Area	Extent of Deprivation by Assembly Area	Mean Age (yrs)	Mean BMI	UE *n* (%)	Retired *n* (%)	Mean MDS (SD)	Knew about Med diet *n* (%)	Would consider making changes to diet *n* (%)
1	5	Male	West Belfast	76	53.8	35	0 (0)	1(20)	2.8 (1.48)	1 (20)	5 (100)
2	7	Female^a^	West Belfast	76	54.7	33^c^	4(57)	0 (0)	1.6 (1.81)	2 (29)	6 (86)
3	5	Male^a^	West Belfast	76	64.6	31	0 (0)	4 (80)	0.6 (0.55)	0 (0)	5 (100)
4	6	Female	Urban mix	–	72	26	1 (17)	5 (83)	4 (1.41)	4 (67)	6 (100)
5	2	Female	West Belfast	76	56	29^c^	1 (50)	0 (0)	3.5 (0.71)	2 (100)	2 (100)
6	5	Female^a^	Holywood	3	56.2	26	1 (20)	0 (0)	2.4 (1.14)	2 (40)	5 (100)
7	6	Male	Urban mix	–	65.8	28^c^	0 (0)	5 (83)	2.7 (0.52)	5 (83)	6 (100)
8	6	Female	Whiteabbey	59	62.5	30	0 (0)	4 (67)	2.3 (1.97)	4 (67)	5 (83)
9	3	Female	Randalstown^b^	5	70.3	28^c^	1 (33)	2 (67)	1.3 (1.16)	1 (33)	3 (100)
10	4	Male	Randalstown^b^	5	59.3	27	2 (50)	2 (50)	1 (0.82)	0 (0)	4 (100)
11	11	Female^a^	Kilcoo^b^	7	75.1	25^c^	5 (46)	5 (46)	2.3^c^ (1.42)	1 (10)	9 (82)
12	7	Male	Urban mix	–	64.6	29	0 (0)	4 (57)	2.3^c^ (1.97)	5 (71)	7 (100)

^a^Indicates a previously existing group. ^b^ Indicates a rural area. ^c^ Indicates that this data was missing for some participants, the average has been calculated from the incomplete data. *UE* unemployed, *MDS* Mediterranean Diet Score out of possible 8 points for high consumption of fish, vegetables, wholegrain, nuts and fruit, moderate consumption of red wine and low consumption of red meat. Extent of deprivation shows the percentage of an area’s population living in the most deprived super output areas in the Northern Ireland [46], therefore higher percentages denote a higher level of deprivation. This could not be calculated for the mixed groups

**Table 2:** Exemplar quotes for theme 2

	Motivating Factors	De-motivating factors
Personal	Reason to change	Inconvenience
	*People would need to be driven to attend those kind of things and if you felt you needed support you’d be more inclined to do it.* FG1 (male, less affluent)	*Once a week? Some of us are busy men.* FG3 (male, less affluent)
		*You see for the likes of us and no transport at night, if things were held in the middle of the day it would be easier for them to get to. .. And then there’s some older people doesn’t like to go out at night.* FG9 (female, more affluent)
	Measurements	Lack of interest
	*I need to be going face to face or getting on a scale with somebody and somebody saying to me, you know, you haven’t lost weight or you have lost weight to spur me on.* FG5 (female, less affluent) *And then the other thing that would be interesting would be for somebody to show evidence that by you changing your diet that it actually has helped your health. If you’re doing that for health and you’re really struggling because you really don’t like but if you feel that when you change and you’re feeling better and the results are coming, that your blood pressure’s coming down or your cholesterol and so on, because everybody needs just a little bit of reassurance that you’re succeeding*. FG8 (female, less affluent)	*I think with discussion groups, they’re okay but they fade out very quickly, especially in bad weather and all the rest of it. Who is going to get up if there’s something on the TV and they’re sitting with a beer or whatever it is, am I going to go into town to this discussion group about Mediterranean food? I think not.* FG1 (male, less affluent)
		*If you maybe sort of feel it’s an awful night, I couldn’t be bothered going, that sort of thing, you can talk yourself out of the things quite often.* FG4 (female)
		*And part of the reason for that is distraction, “oh God, it’s a wet night.” “There’s a big match on the box” whatever. So the motivation, keeping the motivation, really your point puts that slightly differently, having the motivation to get started is one thing, sustaining that motivation and seeing the benefit is another thing.* FG7 (male)
Interpersonal	Support	Poor relationships
	*And the fact that you’ve contacted somebody else who’s going through the same thing as you is good, because if you’ve got the urge to go back or retract then they’re encouraging you on, they’re saying, “stick with it.”* FG9 (female, more affluent)	*I think I would go for a mixture because a group is okay but if you’re there every week sometimes personality clashes come into play, no matter what group you’re in, should it be a sporting group, any kind of group.* FG10 (male, more affluent)
	*Yeah, because there’ll always be things comes up. It happens where you won’t be able to make it 1 week, and then sometimes if you can’t make it 1 week and it happens the next week you can slowly drift away, so if you have a back-up there where you can phone and say “look, I’m not going to be able to get for a few weeks” and just keep in the loop with what’s going on or what’s happening.* FG5 (female, less affluent)	*It depends on the group too. If you get people who are a bit intrusive or going out and sharing things, but generally speaking I think groups meetings are useful.* FG4 (female)
	Accountability	
	*Yeah definitely. You’re getting help with managing it by discussing it with other people and usually in a group you’d be more inclined to think “I’ll give it a go because I don’t want to let myself down in front of everybody.”* FG5 (female, less affluent)	
	*Because you’ve made the arrangement to be there you do go. It’s written in stone.* FG8 (female, less affluent)	
	Competitiveness	
	*It* (telephone support) *wouldn’t be for me because I’m the type of person who if I decide to do it I’m going to do it, and the only thing that would keep me going would be the group and seeing how well they’re doing, and maybe a wee bit of jealously*. FG10 (male, more affluent)	
	*I think if you’re comparing weight and things like that it would motivate you. Say, you lost a pound 1 week and I didn’t, you know that sort of thing.* FG4 (female)	
	Role models/ Peer pressure	
	*Say someone had a good result, that result would help motivate the others to maybe stick to the diet.* FG3 (male, less affluent)	
	*In a group situation there tends to be maybe a slight pressure on you to be seen to conform to what is good, simply because if you go each week then you want to be seen to be successful, and it does work.* FG10 (male, more affluent)

**Table 3:** Peer supporter characteristics

Characteristic	Important? *n* (%)	Males *n* (%)	Females *n* (%)
Similar age to you	16 (25)	7 (28)	9 (23)
Similar gender to you	16 (25)	5 (20)	11 (28)
Lives in the same area as you	15 (23)	5 (20)	10 (25)
Has successfully made the recommended changes to their diet	54 (83)	20 (80)	34 (85)
Is like you and wants to make similar changes to their diet.	46 (71)	18 (72)	28 (70)
Has expert dietary knowledge	47 (72)	17 (68)	30 (75)
Is someone you already know	7 (11)	4 (16)	3 (8)

### Qualitative data collection

Vignettes were used to stimulate conversation and improve understanding of the different peer support methods for participants. Vignettes are concise stories explaining similar characters and backgrounds with only one differing feature under investigation, which in this case was the method of peer support provided. They were developed by the research team based on examples used in previous studies [27–29]. Four different vignettes were utilised, each describing one of the four peer support methods: group, one-to-one, telephone and online. A unisex name was used in the vignettes therefore identifying with both genders during discussions (see Appendix).

Focus group schedules included discussion of dietary change, MD and heart health prior to the introduction of peer support as a method of encouraging dietary change towards a MD. Facilitators introduced each method in turn using the appropriate vignette followed by a discussion stimulated by questions such as: ‘do you think such support as I have just described might be useful in encouraging you to eat a Mediterranean diet?’ and ‘what do you like and dislike about it, and why?’. After all of the methods of peer support had been introduced, the facilitator asked what participants’ preferred method would be or whether a combination of methods could be helpful. Following this there was a brief discussion about the characteristics and skills required of a successful peer supporter. All discussions were tape-recorded and transcribed verbatim with all names changed to pseudonyms to ensure confidentiality and anonymity.

### Qualitative data analysis

Qualitative data were analysed thematically using an inductive approach. Transcripts were read multiple times by CE to facilitate the development of codes indicating deducted themes and sub-themes with a clear focus on what participants preferred and why. The original coding framework was discussed with CMcE and further development of themes was discussed with MMcK. Consideration was given to contradictory or opposing views, and differences in acceptability of peer support methods for different groups. Transcripts were then coded using NVivo 10, a qualitative indexing software package, to manage the data and were further analysed to identify subthemes and illustrative quotations.

## Results

### Participant characteristics

A total of 67 participants took part in focus groups, 27 males and 40 females. Three quarters of the focus groups took place in an urban setting (*n* = 9) and one third (*n* = 3) in rural settings.

The age of focus group participants ranged between 44 and 86 years (mean 64.0, SD = 10.03). The majority of participants were overweight or obese: BMI values ranged from 20 kg/m^2^ to 41 kg/m^2^ (mean 28.8 kg/m^2^, SD = 4.53). MDS ranged across groups from 0.6–3.5 out of a possible total of 8 (mean MD score 2.3, SD = 1.57). Previous awareness of MD ranged from 10 to 100% across groups; 40.9% (SD = 0.50) of the total study population were aware of the MD. Overall, 98.4% (SD = 0.13) of participants considered themselves open to changing their diet.

### Preference scoring questionnaire

**Fig. 1:**
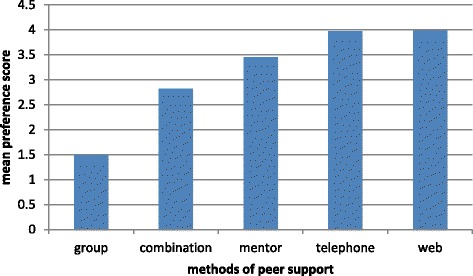
Mean preference scores for different peer support methods. *Ranked score where 1 = most preferred peer support approach to 5 = least preferred peer support approach. ^#^Combination preferences were: Group + telephone; Group + mentor; Group + web

As indicated in Fig. 1, based on the individual preference scoring sheet, the group peer support was the most preferred approach, followed by a combination of methods, a peer mentor approach, and the least preferred options were, equally, telephone and internet based support.

### Thematic analysis of focus groups

It was apparent from the focus group discussions that a group model of peer support was already familiar to many participants, as two commercial weight loss schemes which involve a substantial group peer support component were mentioned in every focus group. One participant mentioned previously taking part in a study involving mentor peer support and another discussed taking part in internet forums for weight loss.

Analysis of the transcripts elucidated some of the reasons why group peer support was the most preferred method. The reasons were encompassed in three themes: components of an effective peer support group, catalysing motivation and stepping stones of change and are described in more detail below. The last theme also included discussion of the merits of having individual choice or using a combination of approaches to deliver peer support.

### Theme 1: Components of an effective peer support group

**Fig. 2:**
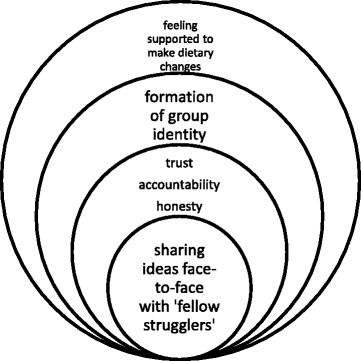
Layers of an effective peer support group

This theme emerged from identification of the predominantly positive discussions surrounding the group peer support approach. Participants identified what they considered to be the useful components that would facilitate an effective peer support group. These are depicted in Fig. 2, each layer of the circle illustrating a necessary component of an effective peer support group, with each layer building upon the one before.

Figure 2 demonstrates that the core elements for an effective peer support group are sharing knowledge with a group face to face. Group peer support was considered preferable to telephone and mentor peer support as it was seen to enable participants to learn more from interacting with a number of people. It was suggested that this would be particularly beneficial in developing problem solving techniques.

*Give people ideas and you learn from other people’s ideas.* FG8 (female, less affluent).

*I think with this sort of approach when you’re getting together with other people you’re able to sort of experience what other people are eating and see then, even swapping dietary advice, cooking tips and things like that there, ways to prepare food, even on a reasonable sort of budget, if you were trying to cook for a family but also for yourself. It would be a lot of knowledge from other people getting together. I always think you learn something which you maybe don’t know.* FG5 (male, less affluent).

Face to face contact was one of the key perceived benefits of group peer support over internet and telephone peer support. It was suggested that this would increase honesty and accountability between peers and increase the perceived level of support.

*I think it’s always better to see a person and have that sort of human contact to know that there is someone on your side. An anonymous voice at the end of a telephone is not as reassuring, and if you have a face to that voice behind the phone, that’s fine as an extra support. But when you think you’ve got somebody in your corner and you’re looking at them and you’re talking to them and that’s helpful; an anonymous person at the end of the phone, no.* FG8 (female, less affluent).

*People that need to make dietary changes and don’t see anyone face to face probably lie [laughter], because I would. “Yes, I’ve been really good I’ve been brilliant!”* FG5 (male, less affluent).

Participants discussed that having face to face contact with a group, and sharing knowledge with each other would help to build trust, honesty and accountability. These elements were discussed as being essential for an effective peer support group.

*It seems to me the effectiveness of the group would depend on two things. One of them would be the extent to which people are able to trust each other and be open and honest about what their habits really are, and secondly, if you’re going back and you know that somebody is going to say to you, “well, what changes have you made?” and you are going to be held to account.* FG7 (male).

In the quotes below, phrases such as ‘collective message’ and ‘fellow strugglers’ indicate formation of a group identity through the group support model. During some focus groups (both those newly formed for the study and those formed from previously existing community groups) it was suggested that the peer support may be particularly effective within existing groups who already have a close bond.

*I think groups are good because not only do they convey a message, a collective message, you get a sense of bringing something together.* FG10 (male, more affluent).

*I’m just trying to imagine it, and I think it’s why I would be more comfortable in a group environment than with this (peer mentor approach), I think in a group environment you’re with a bunch of, if you like, fellow strugglers.* FG7 (male).

Theme 1 brings together the explanations offered by participants for their preference for group support to identify the necessary components of a successful peer support group.

Discussion regarding peer support groups was predominantly positive, although there was some mention of the potential that it may be inconvenient due to its time, date, location or commutability. There was also some discussion that group support may not be suitable for everybody as people may not feel comfortable in group situations. This issue is discussed further in theme 3.

### Theme 2: Catalysing motivation

Table 2 provides exemplar quotes for this theme. Participants discussed how motivation was an important factor in helping people to change to a MD and that different models of peer support might provide different types and levels of motivation. Participants felt that the group model of peer support would use interpersonal factors to catalyse motivation through support and encouragement between members, peer pressure, competitiveness and feeling accountable. Measurements of weight or blood pressure at group meetings were also seen to provide evidence of success, which participants also considered to be motivating.

Participants discussed how individuals would need to have an initial motivation to attend the peer support group, such as being overweight, having high blood pressure or cholesterol or having a friend or family member who had suffered heart disease. Likewise, they acknowledged the challenges of sustaining motivation such as having a busy lifestyle, being distracted by television, not wishing to travel in poor weather or having difficulty forming positive relationships within the group.

### Theme 3: Stepping stones of change

Theme 3 (stepping stones of change) comprised discussions about how a combination of methods may be the most effective way of encouraging behavioural change and maintenance. It was evident in discussions that, whilst group peer support was overall the preferred approach, participants also felt that other methods of peer support might be useful at different points in time or for different individuals. Participants conceptualised change as a process, and considered that, at the beginning of the process, more intense support might be required such as would be provided by the peer mentor approach. They suggested that this could be followed by face to face group support and that internet or telephone approach could be utilised to assist in the final stages or maintenance of change depending on personal preference.

Mentor peer support was seen as the most intensive intervention and it was suggested that this may be most useful when a person was starting the process of changing as well as for those who dislike attending groups.

*The one to one is nice but it could be a bit intense after a while.* FG8 (female, less affluent).

*But I think, getting back to this whole situation, it does boil down to group support, and for some for a period of time maybe one to one just to keep you going. When you’re starting something new you need encouragement and that is when you might need it and then when you get going, if that person who you had as a one to one would always be at the end of the phone line if you needed to contact them to say help.* FG8 (female, less affluent).

*I think it very much depends on the individual. If you get a real shrinking violet they’ll not want a group session, whereas us four here, we can all talk the best and amongst each other but it’s what an individual’s character, what their makeup is too.* FG10 (male, more affluent).

Participants suggested that a group-based approach was the most preferable method to follow mentor peer support. To sustain change, it was considered useful to have a website to refer back to or a peer supporter to call when experiencing difficulties.

*It’s like a personal trainer, I suppose, that type of idea. But I think the group idea is better than the one to one, I would prefer that myself, because if it’s a one to one it’s complete focus on each other and I don’t know, it just wouldn’t … I would prefer the group, I wouldn’t like the one to one basis, I wouldn’t really.* FG4 (female).

*I think the one to one to start with and then the group and maybe then that site that I can dip in and out of, because if shops ask me for my email and things like that I just couldn’t be bothered receiving all those. But if I knew there was a site there that I could go into and get some bits and pieces then, yes, I would do that.* FG6 (female, more affluent).

*Yeah, I like being in a group session and looking up a website and seeing different menus, easy steps to follow through.* FG12 (male).

*The group’s the best. Through the group you can expand all sorts of options, you could have ones meeting up for coffee to see how they’re getting on. If you had to phone Joe Bloggs, you know who Joe Bloggs is and what they look like and you know what they’re talking about, but not a complete anonymous voice.* FG8 (female, less affluent).

Participants also suggested that different people may require or prefer different models, so having the option of when to move to each model or which to use might be very useful.

*Surely the lesson of life is that it’s neither one nor the other that might work but it’s usually a combination of things.* FG10 (male, more affluent).

*I sort of see a combination developing for me, just going through my head listening to the scenarios and different things. Initially you start off in a group. Some people are that wee bit more sort of confident and then can go off and do their own thing, some people aren’t just as confident maybe, they’re new to it and they need to learn skills, give them that extra help. And then the third step being … you were talking about links with computer, email and different things and then sort of stem off for that. So people get what they need out of it; if you need a lot you can get a lot.* FG1 (male, less affluent).

### Format and content of peer support groups

There was some discussion during focus groups about how the face-to-face peer support groups could look from a more practical perspective. There was preference for an informal environment, such as a community centre. Weekly or monthly meetings were suggested as the ideal frequency, with some participants suggesting a tapering of the frequency may be helpful to achieve dietary change.

*I would say you probably need more meetings at the start … to change and then less after that. But initially you would need more. FG6 (female, more affluent).*

The sharing of personal experiences was considered useful for making dietary changes, this has been discussed in theme 1. Practical sessions, such as cookery demonstrations, were also considered useful.

Measurements at group sessions were discussed as being helpful in that they could provide an indication of success and also provide a justification for changing diet.

*If someone’s very much overweight then there’s a good chance that there’s a greater risk of heart disease. I think that’s well accepted in society. If you’re overweight you’re putting more pressure on your heart, and so I think they already have an indicator. But someone like myself, I’m not skinny and I’m not overweight but I’m somewhere in between, people would be saying to me ‘what do you want to do that for? You’re all right.’ But if I had some kind of indicator that said ‘oh...’ then I might say ‘yeah, definitely, this is a good idea. Go for it.’* FG10 (male, more affluent).

Male focus group participants discussed that it could be beneficial to bring a partner to the group meetings, particularly if their partner was the main cook in the household.

*If there was somebody living long term in the house you would want them involved for moral support as much as anything else.* FG10 (male, more affluent).

### Exploration of peer supporter characteristics

Focus group participants were asked whether the background, age and gender of peer supporters would be an important factor in a questionnaire (Table 3). The majority did not think it was important that the peer supporter would be a similar age (75%), gender (75%) or from the same area (77%). Rated most important were that the peer supporter would have successfully made changes towards a MD (83%), they would have expert dietary knowledge (72%) and the group members would feel the peer supporter is like them and wanted to make similar dietary changes to them (71%).

Focus group discussions revealed that a good peer supporter should be empathetic, encouraging and have a good sense of humour, plus they should have personal experience of eating a MD, good knowledge of health and budgeting and strong communication and listening skills. See Table 4 for exemplar quotes regarding preferred qualities, attributes and skills of peer supporters.

**Table 4:** Peer Supporter attributes

Subject	Theme	Illustrative Quote
Qualities	Empathetic	*Non-judgemental. So if you don’t succeed they’re not on your back and you know, “this is really what you should be doing” or you know, but just sort of be gentle. FG2*
	Encouraging	*You find a lot of these groups, WeightWatchers and some of these, the ones that are very popular are with somebody who is very good at taking the group and really is very motivated and motivates everybody else.FG4*
	Sense of Humour	*Has to be able to take a joke*. *FG3*
Attributes	Personal Experience	*I think you need a role model. You need someone that you can say, “well, if they’ve done it I can do it” and someone who’s been there will understand the pitfalls and give you good support. FG8*
	Knowledge	*I think the supporter needs to have a basic understanding of health issues, because there’s no use Glenda and I, me saying to Glenda, “oh you just have to do this and your blood pressure will go down. Mine is down.” There’s obviously something different between your genetic makeup and mine that my blood pressure never goes up, and my husband’s on blood pressure tablets. So there’s no point me trying to support Glenda and saying “do this, do this because it worked for me.” There has to be a basic understanding of health issues and various things. FG4*
		*They are tuned in to people’s needs money wise and stuff, it’s not somebody coming from Malone [affluent area of Belfast] saying “oh you should have this, this and this” when really that’s not in people’s budgets, it’s not realistic. FG2*
Skills	Communication	*They have to be able to communicate the message in a convincing way. FG10*
	Active Listening	*Somebody who listens and would have empathy but he or she doesn’t have to have anything in common, as long as you could have a rapport. FG1*

## Discussion

### Main findings and interpretation

Focus group discussions in a Northern European population at high risk of CVD found that there was a strong preference for group peer support to encourage adoption and maintenance of a MD.

This study is one of few in the literature to employ a thorough approach to peer support intervention design, allowing the target group to shape its format, content and delivery and contributing to behaviour change literature. The ‘Football Fans in Training’ Intervention [30] developed an expert multidisciplinary team to decide on their preferred method of peer support delivery. Although it focused on changing physical activity patterns rather than diet, there are some key similarities to our findings; aiming to focus on practical and experiential learning which authors believe strongly contributed to its success.

Theme 1 identified the predominantly positive viewpoint taken in discussing the group peer support model and how participants considered some of the key ingredients for an effective peer support group. One of the key ingredients for an effective peer support group was to the opportunity to form a group identity, this has been discussed as important in literature [31–33]. Interestingly, group identity is often discussed in the literature in accordance with groups developing a ‘sense of ownership’. Such an issue was not discussed by participants during focus groups in this study, where the focus was more on the support, commonality with other participants and motivational aspects gained by giving and receiving support in a group setting.

The benefits of group peer support in catalysing motivation have also been found in a study of mental wellbeing in which group members reported feeling ‘uplifted by exchanging emotional and practical support; they gained self-esteem, knowledge and confidence, thereby increasing their control over their situation’ [34]. Similar suggestions of challenges to motivation, including inconvenience, lack of interest and poor relationships, were found in the literature [35].

The results of this study demonstrate clear preference for a group peer support approach. However, the findings reported under Theme 3 suggest that a combination of peer support approaches may be most likely to suit the widest range of people. Other research teams have faced the challenge of developing an intervention suitable for a number of people, one utilised telephone support for people with diabetes and found that some participants preferred to speak to a diabetes nurse than a peer [36]. Other studies have found that a structured group-based support does not appeal to all patients or populations, and that drop-in groups or telephone contact may work better for those participants [21, 32, 37]. In this study, participants also discussed the challenges of constructing an intervention that could serve everyone, and recommended a combination approach, involving a group support component. Due to the complexity of behaviour change, it is prudent to test one method thoroughly; the process evaluation of which will determine whether additional peer support methods are required. Each additional layer of approach is likely to incrementally increase complexity and cost, and these are important considerations for implementation of public health interventions.

The peer supporter role has been discussed in the literature with regards to the need for training, the personal benefits to peer supporters of participating and the benefits that a peer supporter with personal experience of relevant issues brings to the role [38–42]. This study adds further information regarding the qualities, attributes and skills that a population at high risk of CVD consider to be beneficial in peer supporters. Key necessary characteristics identified by participants were empathetic, encouraging, having personal experience of MD, having knowledge of disease risk factors and the MD and having strong communication and listening skills. It was considered less important that the peer supporter would be a similar age or gender. This highlights the question of how best to recruit and also train peer supporters, to get a pool of people with these characteristics.

### Strengths

A particularly strong aspect of this study was the inclusion and exclusion criteria, as this helped to ensure that the population would possess similar characteristics to future intervention participants. Furthermore it is valuable that the focus group participants were of different genders, varying levels of affluence and from urban and rural areas as this ensured a range of opinions were considered. There were no clear differences between participants differing in the aforementioned characteristics.

Vignettes were used in this study to help participants ‘visualise’ the different peer support approaches and encourage discussion. This may be particularly important when participants have little prior experience of the concepts or scenarios being discussed. The use of vignettes in qualitative and quantitative research is reasonably common throughout a number of disciplines. The vignette scenarios must be considered authentic to be effective and aim to reduce limitations of participants personal experience or circumstances [43, 44]. It has been suggested that vignettes are limited in their effectiveness as the relationship between belief and action is not often straightforward. It was considered in this study that they would be beneficial as an ‘icebreaker’ to stimulate discussion, and their use in conjunction [45] with a structured interview would mediate any negative aspects.

### Limitations

The differing familiarity of different support methods could perhaps be seen as a weakness to the study. ‘Weightwatchers’ and ‘Slimming World’ were regularly discussed as reference points which may have introduced familiarity bias in discussions. It may be possible that a group method received the most favour as it was the most familiar and readily understood method of peer support, although these effects were potentially lessened by the use of vignettes.

It could be suggested that collecting data using focus groups, a face-to-face group setting, may have influenced the preference for face-to-face group peer support. Participants may have enjoyed taking part in the focus group discussion which could have influenced their decisions in the preference scoring sheet, administered at the end of the focus group. It could also be possible that those who accepted invitations to participate in a focus group would be more likely to prefer a group method of peer support or that recruitment of people from existing community groups inadvertently recruited those who particularly enjoy participating in face-to-face group formats. However the use of focus groups was key to access group beliefs about the different methods of peer support.

### Future research directions

This research has been used to inform the development of TEAM-MED, a MD peer support intervention. It will also be valuable during the final evaluation of the intervention as it may be useful to explore whether the perceived benefits of group peer support were apparent or whether any particular types of motivation were identified as more or less useful by participants.

Further studies are recommended into effective peer supporters, the development and optimisation of an appropriate training programme for peer supporters and to explore the possibility and benefits of utilising existing groups to deliver a lifestyle behaviour change intervention, rather than ‘creating’ groups for this purpose. In line with the MRC framework for the development and evaluation of complex interventions [23], this work highlights the value of conducting exploratory research with the target group to inform intervention design, and provides important information on preferred peer support approaches in a Northern European population that will be useful to other researchers who plan to develop behaviour change interventions.

## Conclusions

This research demonstrates that a group peer support approach is most likely to be acceptable to encourage adherence to a MD in a Northern European population at high risk of cardiovascular disease. Participants discussed that commonality between group members which would lead to formation of a group identity and developing trust, honesty and accountability. It was suggested that this model could increase motivation through support, accountability, competitiveness and providing role models or peer pressure. Further potential benefits were suggested from using a combination of peer support methods. Participants identified key characteristics for peer supporters to be empathetic, having personal experience of MD, being knowledgeable and having strong communication and listening skills. The findings have been used to inform the development of a MD intervention which will be piloted in the study population.

## Appendix

### Vignettes for focus group participants

Chris has a higher risk of developing heart disease and stroke and has been advised to make dietary changes to improve this risk. We are interested in the type of support needed to help her/him make the recommended changes.

Have a look at these examples showing how Chris could be supported to make dietary changes. Assume that Chris has more or less the same age, education and work history that you have. Other than the conditions mentioned you should imagine Chris is in reasonably good health. Tell us whether the support being offered will work or not – and why you think the suggestions are good or bad.

Group meetings

1. Chris meets up regularly with a group of people who are also at high risk of developing heart disease, and trying to make changes to their lifestyle. During the group meetings, Chris learns about food that will help her/him to manage her/his health risks and finds out how everyone else is getting on with making dietary changes. By discussing these things in a group, Chris gets help with setting her/his own goals and also support and encouragement from others in a similar position to herself/himself.

Personal Mentor

2. Sam successfully managed to reduce her/his risk of heart disease by adopting the dietary changes that were suggested to her/him. She/he learnt a lot from her/his own experience and wanted to use this knowledge to help other people. So these days Sam is in contact with Chris, on a one-to-one basis to provide ongoing encouragement and support to help Chris to make similar dietary changes. When they meet, Sam asks how things are going, discusses any problems or concerns that Chris has about her/his diet and offers Chris advice by drawing on her/his own personal experiences of adopting a healthier diet.

Telephone help

3. Alex helps people to make lifestyle changes to reduce their risk of developing heart disease. She/He speaks to Chris regularly by telephone and chats about the recommended dietary changes. Although they never meet face-to-face, Alex provides ongoing encouragement, support, motivation and confidence to help Chris overcome any difficulties she/he may have in making the dietary changes that have been recommended to her/him.

On-line

4. Chris receives regular emails and text messages providing support, encouragement and motivation to help her/him make the recommended dietary changes. Chris has also joined an online discussion forum to communicate with other people who are trying to make similar dietary changes. In this way, she/he is able to share her/his personal experiences and provide some support and motivation to others as well as getting the advice and help that she/he needs for herself/himself.

## Abbreviations

CVD: Cardiovascular disease
FG: Focus group
JBS: Joint British Society
MD: Mediterranean diet
MDS: Mediterranean diet score
ORECNI: Office for Research Ethics Committees Northern Ireland
RCT: Randomised control trial
TEAM-MED: Trial to Encourage Adoption and Maintenance of a MEditerranean Diet
